# Supplemental wheat germ modulates phosphorylation of STAT3 in the gut and NF-κBp65 in the adipose tissue of mice fed a Western diet

**DOI:** 10.1016/j.cdnut.2022.100023

**Published:** 2022-12-23

**Authors:** Babajide A. Ojo, Sanmi E. Alake, Amritpal Kaur, Siau Yen Wong, Bryant Keirns, Jerry W. Ritchey, Winyoo Chowanadisai, Dingbo Lin, Stephen Clarke, Brenda J. Smith, Edralin A. Lucas

**Affiliations:** 1 Division of Pediatric Gastroenterology, Department of Pediatrics, Stanford University School of Medicine, Stanford, CA, USA; 2 Nutritional Sciences Department, Oklahoma State University, Stillwater, OK, USA; 3 Department of Veterinary Pathobiology, Center for Veterinary Health Sciences, Oklahoma State University, Stillwater, OK, USA; 4 Department of Obstetrics and Gynecology, Indiana University School of Medicine, Indianapolis, IN, USA

**Keywords:** adipose tissue, gut-adipose axis, antimicrobial peptides, high-fat diet, inflammation, macrophages, Western diet, wheat germ

## Abstract

**Background:**

Commensal gut bacteria, including *Lactobacillus,* can produce metabolites that stimulate the release of gut antimicrobial peptides (AMPs) via the signal transducer and activator of transcription (STAT)3 pathway and prevent obesity-associated leaky gut and chronic inflammation. We have previously reported that wheat germ (WG) selectively increased cecal *Lactobacillus* in obese mice.

**Objectives:**

This study investigated the effects of WG on gut STAT3 activation and AMPs (Reg3γ and Reg3β) as well as the potential of WG to inhibit nuclear Nf-κB–activation and immune cell infiltration in the visceral adipose tissue (VAT) of mice fed a Western diet (i.e., high-fat and sucrose diet [HFS]).

**Methods:**

Six-wk-old male C57BL/6 mice were randomly assigned to 4 groups (*n* = 12/group): control (C, 10% fat and sucrose kcal) or HFS (45% fat and 26% sucrose kcal) diet with or without 10% WG (wt/wt) for 12 wk. Assessments include serum metabolic parameters jejunal AMPs genes, inflammatory markers, and phosphorylation of STAT3 as well as VAT NF-κBp65. Independent and interaction effects of HFS and WG were analyzed with a 2-factor ANOVA.

**Results:**

WG significantly improved markers of insulin resistance and upregulated jejunal *Il10* and *Il22* genes. The HFS + WG group had a 15-fold increase in jejunal pSTAT3 compared with the HFS group. Consequently, WG significantly upregulated jejunal mRNA expression of Reg3γ and Reg3β. The HFS group had a significantly higher VAT NF-κBp65 phosphorylation than the C group, while the HFS + WG group suppressed this to the level of C. Moreover, VAT *Il6* and *Lbp* genes were downregulated in the HFS + WG group compared with HFS. Genes related to macrophage infiltration in the VAT were repressed in the WG-fed mice.

**Conclusion:**

These findings show the potential of WG to influence vital regulatory pathways in the gut and adipose tissue which may reduce the chronic inflammatory burden on these tissues that are important targets in obesity and insulin resistance.

## Introduction

Adipose tissue dysfunction in obesity is characterized by macrophage infiltration and activation which results in chronic inflammation that contributes to obesity comorbidities, such as insulin resistance [[1], [2], [3]]. Indeed, animal and clinical studies suggest that obesity disrupts intestinal tight junctions (TJs), allowing for the increased translocation of bacteria and endotoxins (termed bacteremia and endotoxemia, respectively) from the gut into the blood and surrounding tissues that initiates inflammation and insulin resistance [[4], [5], [6], [7], [8]].

In response to bacterial endotoxin and proinflammatory stimuli, lipopolysaccharide binding protein (LBP) is produced in the hepatocytes and adipocytes which increases the severity of toll-like receptor 4 (TLR4) and Nf-κB–orchestrated inflammation by several folds [[9], [10], [11], [12], [13]]. Although LBP production may be a beneficial physiologic response in cases of acute infection [9, 12], the chronic nature of the obesity-induced leaky gut may continually expose the peripheral tissues to endotoxin leading to local and systemic chronic inflammation associated with obesity and insulin resistance. As such, LBP may be a marker of obesity-induced insulin resistance even in humans [14]. Therefore, strategies that suppress LBP expression in the liver or adipose tissue may alleviate inflammation and insulin resistance in obesity.

Out of the TJ proteins responsible for the maintenance of the gut intestinal barrier, the pore-forming CLAUDIN 2, has received increased attention in recent years [[15], [16], [17], [18], [19]]. CLAUDIN 2 is a paracellular cation-water channel mainly expressed in leaky epithelia and impacts its sealing potential and TJ ultrastructure [15, 16]. Hence, an increase of CLAUDIN 2 in the gut is associated with several diseases, including inflammatory bowel diseases, infectious diseases, and cancer [15]. Additionally, upregulation of CLAUDIN 2 is observed in the small intestine of high-fat (HF)-fed animals, suggesting an involvement in obesity-induced gut dysbiosis and permeability [16, 17]. It is noteworthy that the permeability across the intestines may be dependent on region and time [18]. Among intestinal sections, jejunal permeability was elevated over time in HF-fed animals [18] and in humans with obesity [19]. Therefore, mitigating permeability in the jejunum may be vital to suppress the inflammatory consequence of bacteremia and endotoxemia in peripheral tissues.

To combat systemic inflammation that may be initiated by the epithelial passage of both commensal and pathogenic bacteria, the mammalian gut develops a number of defense mechanisms. Among these are the antimicrobial peptides (AMPs), such as defensins, cryptidins, and the regenerating islet-derived protein (Reg)3 lectins, chiefly produced from the Paneth cells in the intestinal crypts [20]. Interestingly, certain commensal bacteria show immunoprotective ability in the gut by metabolizing dietary factors which stimulate the release of AMPs. For example, gut bacteria-derived butyrate and tryptophan metabolites from *Lactobacillus* protect intestinal integrity by elevating the expression of IL-10 and IL-22 capable of activating the signal transducer and activator of transcription (STAT)3-Reg3 pathway in the intestinal epithelial cells [21, 22]. Consequently, an increase in gut Reg3 lectins prevents bacterial translocation to peripheral tissues [23]. Thus, it could be hypothesized that dietary approaches that enhance the production of AMPs in the gut may reduce the impact of high-fat (HF)–induced bacteremia/endotoxemia and suppress inflammation in peripheral tissues.

Accumulating evidence from our group and others shows that certain whole foods containing a variety of nutrients possess gut modulatory and prebiotic effects in both animals and humans [[24], [25], [26], [27]]. For example, wheat germ (WG) is a nutrient-dense component of the wheat grain that contains about 4% fiber, 23% protein, and several bioactive compounds, including phytosterols, tocopherols, policosanols, carotenoids, and thiamin with various health benefits [28, 29]. Apart from its fiber component, WG is a rich source of the essential amino acid, tryptophan, which could be available for the metabolic survival of certain commensal gut bacteria, including members of the *Lactobacillus* genera [30, 31]. Interestingly, WG feeding showed potential to promote healthy gut bacteria in animals and in humans [26, 32]. We have previously shown that when mice fed a 60%-fat diet for 12 wk, WG supplementation selectively elevated the commensal gut bacterial family *Lactobacillaceae*, reduced serum profile of proinflammatory cytokines (tumor necrosis factor-α [TNF-α], IL-6, IL-1β, and IL-17), and decreased markers of insulin resistance [26, 29].

Despite these reported health benefits, it is unclear whether WG could activate the STAT3-AMP pathway in the jejunum and influence adipose tissue inflammation observed in obesity-induced insulin resistance, especially using a Western diet model. This is important because dietary factors that induce gut AMPs may abrogate the translocation of gut-derived antigens and suppress the initiation of inflammation in the peripheral organs [23]. Therefore, the aim of this study was to investigate the effect of WG on STAT3 activation and AMP expression in the jejunum, and its potential to reduce inflammation and immune cell infiltration in the visceral adipose tissue (VAT) of C57BL/6J mice fed a control or a Western diet for 12 wk. We *hypothesized* that WG would activate STAT3 in the jejunum and increase the transcription of Reg3 lectins, indicating an enhanced capacity to mitigate HF–diet-induced leaky gut. This effect of WG on the gut would be associated with reduced inflammation and immune cell infiltration in the VAT.

## Methods

### Animals and treatment groups

Animal care was carried out at Oklahoma State University Laboratory Animal Research facility maintained under humidity- and temperature-controlled conditions and a 12-h light–12-h dark cycle. The experimental protocol was approved by the Institutional Animal Care Committee of Oklahoma State University, and all procedures followed the approved protocol.

Six-wk-old male C57BL/6 mice (*n* = 48) from Charles River Laboratory (Wilmington, MA) were acclimated for a week and randomly assigned to dietary treatment groups (*n* = 12/group) in a 2 × 2 factorial design: control (C; 10% fat and sucrose kcal; AIN-93 M), C + 10% WG (C + WG), a Western-styled diet with high-fat and sucrose (HFS) (45% fat and 26% sucrose kcal), and HFS + 10% WG (HFS + WG) for 12 wk. Mice were group-housed (4 mice per cage) in wire-bottom cages to prevent coprophagy. Food and water were consumed *ad libitum* for 12 wk. Food intake per cage was monitored daily, and the body weight of each mouse was measured weekly.

Shawnee Milling Company graciously provided the germ from Oklahoma red winter wheat. WG was analyzed for its nutrient composition (NP Analytical Laboratories) and added to the control (C + WG) and HFS (HFS + WG) diets at a level of 10% wt/wt. Diet composition and the WG dose were based on our previous study that investigated the effect of WG supplementation on the gut microbiome (Table 1) [26].

**TABLE 1:** Diet composition (g/kg)

Ingredients	C^1^	C + WG^1^	HFS^1^	HFS + WG^1^
Wheat Germ (WG)^2^	—	100	—	100
Carbohydrates				
*Total*	721	721	466.1	466.1
Cornstarch	466	413	34	0
Sucrose	100	100	308.6	308.6
Dextrinized cornstarch	155	155	123.5	106.9
WG^2^	—	50.6	—	50.6
Protein				
*Total*	140	140	172.8	172.8
Casein	140	116.6	172.8	149.2
WG^2^	—	23.6	—	23.6
Fat				
*Total*	40	40	238.3	238.3
Soybean oil	40	32.3	49.4	41.69
Lard	0	0	188.9	188.9
WG^2^	—	7.71	—	7.71
Fiber				
*Total*	50	50	61.7	61.7
Cellulose	50	46.9	61.7	58.6
WG^2^	—	3.1	—	3.1
Vitamin mix^3^	10	10	12.3	12.3
Mineral mix^4^				
*Total*	35	35	43.2	43.2
Mineral mix (Ca-P def)	13.4	13.4	16.54	16.54
Calcium carbonate	12.5	12.4	15.4	15.3
Calcium from WG^2^	—	0.06	—	0.06
Sodium phosphate	5.6	3.48	6.91	4.78
Potassium phosphate	2.4	1.49	2.96	2.04
Phosphorous from WG^2^	—	0.93	—	0.93
Sucrose	1.1	3.29	1.36	3.57
L-Cystine	1.8	1.8	3.1	3.1
Choline bitartrate	2.5	2.5	2.2	2.2
Tert-butylhydroquinone	0.01	0.01	0.01	0.01
Kcal/g	3.96	3.96	4.62	4.62

1 C, control diet; C+WG diet, C+10% wheat germ (WG) diet; HFS, high-fat and sucrose diet; HFS+10% WG diet. WG diets were adjusted to have the same macronutrients, calcium, and phosphorus concentrations to either C or HFS diets.

2 WG composition (Shawnee Mills) analyzed by NP Analytical Laboratory: carbohydrates, 50.6%; protein, 23.6%; fat, 7.71%; fiber, 3.10%; calcium, 0.056%; and phosphorus, 0.93%.

3 Harlan-Teklad Laboratories (TD 94047).

4 Complete mineral mix (TD94049, Harlan-Teklad Laboratories) was used for the C diet and a calcium and phosphorus deficient mineral mix (Ca-P def, TD 98057, Harlan-Teklad Laboratories) was used for the C + WG and the HFS diets.

### Fasting glucose, glucose tolerance test, sample collection, and processing

For fasting blood glucose measurements, mice were feed deprived for 6 h at baseline (wk 0), mid (wk 6), and final (wk 12), and glucose was measured from the tail blood using ReliOn Confirm blood glucose monitoring system (Walmart). At d 80, a glucose tolerance test (GTT) was conducted in mice that were fasted for 6 h as we have previously described [33].

After 12 wk of dietary treatment, mice were feed deprived for 4 h and anesthetized using a mixture of ketamine and xylazine (100 mg and 10 mg per kg body weight, respectively). Body composition was assessed using a whole body densitometer (Piximus, GE Lunar). Blood was collected from the carotid artery and serum was processed as described previously [29]. The small intestine was flushed with ice-cold PBS and excised into 3 different sections. The jejunum (mid-section) was snapped-frozen in liquid nitrogen and stored at −80 °C for further protein and gene expression analyses. The ileum mucosa was collected as previously described [26]. The liver, perirenal adipose tissue, pancreas, and spleen were collected, weighed, snapped-frozen and stored at –80°C for further analyses. The VAT was collected, weighed, and a section was snapped-frozen. In preparation for isolating stromal vascular fraction, a section of VAT was dissected and transferred into an ice-cold tube containing complete medium (DMEM + 10% FBS + 1% penicillin/streptomycin, 1g fat/10 mL) for further analyses.

### Serum measurements

Serum adipokines, hormones, and lipids were determined as previously described [29]. Briefly, total cholesterol and TGs were measured using a Biolis 24i automated chemistry analyzer (Carolina Chemistry). Insulin, leptin, and resistin were measured as part of the Bio-Plex mouse diabetic markers kit (product no. 171F7001M) using the Bio-Plex MAGPIX Multiplex reader (Bio-Rad Laboratories Inc) following the manufacturer’s instructions. The HOMA-IR was used as a surrogate measure of insulin resistance using the formula: HOMA-IR = fasting insulin (μU/ml) × fasting glucose (mg/dl)/405.

### Isolation of adipose-derived stromal vascular fraction

The isolation of adipose-derived stromal vascular fraction (ADSVF) was obtained by enzymatic isolation as described previously with few modifications [34]. In brief, the VAT was diced into fine pieces and incubated at 37°C in 10 mL digesting medium containing DMEM + 1% FBS + 0.2% collagenase type VIII (Sigma, #C2139) while shaking at 300 rpm for 30 mins. The cell suspension was filtered through a 100 μm cell strainer (VWR, #10199-658) into a 50 mL tube containing a complete medium (DMEM + 10% FBS + 1% penicillin/streptomycin). The cell suspension was centrifuged (5 min, 1000 rpm), and the pellet containing the stromal vascular fraction was incubated with 0.83% NH_4_Cl for 4 mins on ice to lyse the red blood cells. The cell pellets (ADSVF) were washed twice in Dulbecco’s phosphate-buffered saline (Sigma, #D5652). Radioimmunoprecipitation assay buffer containing 0.5% protease and phosphatase inhibitor cocktails (Sigma, #P8340 #P0044) was added to the ADSVF (70 μL per 3 million cells) for the preparation of total protein following standard procedures.

### Gene expression analyses

Total RNA was processed from the jejunum, ileum mucosa, and adipose tissue using Trizol reagent (ThermoFisher) following the manufacturer’s instructions. Relative abundance of genes-encoding AMPs (*Reg3β* and *Reg3γ*), *LBP*, cluster of differentiation (*Cd*)*14, TLR4,* inflammatory cytokines (*TNFα, IL6, IlLβ,* interferon [*Ifn*]*γ,* transforming growth factor [*Tgf*]*b1, IL22, and IL10*), innate and adaptive immune cell markers (*Cd11c, F4/80,* C-C motif chemokine ligand [*Ccl*]*2, Ccl3,* inducible nitric xoide syntase [*iNos*], arginase [*Arg1*], *Cd3e,* forkhead box protein [*Foxp*]*3*), hypoxia marker (hypoxia inducible factor 1 subunit α*,* [*Hif1α*]), angiogenesis and adhesive protein markers (vascular endothelial growth factor α [*Vegf*α], intercellular adhesion molecule molecule [*Icam1*], vascular cell adhesion molecule [*Vcam1*]), leptin precursor (*Lep*), and adipose TG lipase (*Pnpla2*) were evaluated using SYBR Green chemistry on an ABI 7900HT system (Applied Biosystems) as previously described [25]. Data were normalized to the cyclophilin gene. The primer sequences used in this study are presented in Supplemental Table 1.

### Protein expression analyses

Total protein extraction and immunoblotting (*n* = 5–6 mice per group) were performed as described previously with few modifications [35, 36]. Total protein homogenates from the jejunum (20 μg), VAT (20 μg), and ADSVF (15 μg) were separated electrophoretically using SDS-PAGE and transferred to PVDF membranes (ThermoScientific). Membranes were blocked with 5% non-fat dried milk (Nestle) at room temperature for 1 h. The membranes were washed twice in PBS and incubated overnight at 4°C in 5% BSA containing the following primary antibodies: CLAUDIN 2 (#sc-293233, Santa Cruz Biotechnology), OCCLUDIN (#33-1500, ThermoFisher), STAT3 (#4904, Cell Signaling Technology), p-STAT3 Tyr705 (#9145, Cell Signaling Technology), NF-κBp65 (#Ab7970, Abcam), pNF-κBp65 Ser536 (#sc-136548, Santa Cruz Biotechnology), FOXP3 (Santa Cruz Biotechnology #sc-53876), and GAPDH (#60004-1, Proteinech). Blots were then washed twice with PBS and incubated with an anti-rabbit (#7074, Cell Signaling Technology), or anti-mouse (#7076, Cell Signaling Technology) IgG HRP-linked antibody. Blots were washed again with PBS, and incubated with SuperSignal West Femto Maximum Sensitivity Substrate (#34095, ThermoScientific) for approximately 1 min. The blots were viewed with FluorChem R Imaging System (ProteinSimple), and the resulting protein bands were quantified with Image J software, v 1.8.0, and the quantified data were adjusted using GAPDH as the loading control.

### Statistical analyses

Statistical analyses followed the use of 2-factor ANOVA (factors of HFS and WG). Least squares means were calculated using the mixed model procedure followed by Tukey’s post hoc test when the *P* value for interaction was significant. A 2-factor repeated-measures ANOVA was carried out on the GTT data using the Huynh-Feldt model. Statistical analyses were conducted using SAS software (version 9.4; SAS Institute). Data are presented as means ± SEM, and a *P* value of <0.05 was considered statistically significant. *P* values for the main effect of diet (control compared with HFS, P_HFS_) and WG (with or without WG, P_WG_) were only displayed on the figures if there was a statistically significant (*P* < 0.05) difference. Whenever a significant *P* value (*P* < 0.05) for interaction (comparing all 4 groups, P_HFS x WG_) was observed, differences between groups were indicated with letters.

## Results

### WG had minimal effects on body weight, tissue weights, and body composition

At the end of the 12-wk dietary treatment, WG supplementation had no effect on the HFS-induced weight gain (Table 2). The HFS-fed group consumed approximately 9% more kilocalories daily than the C group (*P* = 0.022), whereas WG addition in the HFS + WG group reduced caloric intake to the level of C (Table 2). In addition, WG supplementation increased lean mass by at least 2% (*P*_*WG*_ = 0.039) but had no effect in reducing percent body fat and visceral fat (Table 2). WG supplementation had no effect on the relative weights of the liver, spleen, and perirenal fat (Table 2).

**TABLE 2:** Energy intake, body weights and composition, and relative tissue weights of C57BL/6 mice fed a control or high-fat and sugar diet supplemented with or without 10% wheat germ for 12 wk

	C	C + WG	HFS	HFS + WG	*P* value		
					HFS	WG	HFS × WG
Energy intake, *kcal/d*	12.77 ± 0.28^b^	13.62 ± 0.40^ab^	13.97 ± 0.26^a^	12.77 ± 0.16^b^	0.54	0.54	0.0008
Body weights, *g*							
Initial	22.58 ± 0.37	22.77 ± 0.40	22.79 ± 0.30	22.57 ± 0.31	0.99	0.95	0.55
Final	32.66 ± 0.94	32.86 ± 1.05	35.63 ± 1.14	35.20 ± 1.25	0.022	0.92	0.78
Body composition							
Lean mass, *g*	22.70 ± 0.59	24.91 ± 0.77	23.84 ± 0.57	24.34 ± 0.62	0.66	0.039	0.19
Fat mass, *g*	10.79 ± 0.91	8.58 ± 1.11	12.11 ± 1.08	12.26 ± 1.51	0.044	0.39	0.33
Body fat, *%*	31.81 ± 2.19	25.23 ± 2.74	33.17 ± 2.05	32.18 ± 2.98	0.11	0.14	0.28
Relative tissue weights, *mg/g body weight*							
Liver	48.82 ± 1.43	50.77 ± 1.81	41.69 ± 0.86	41.3 ± 1.18	<.0001	0.56	0.39
Spleen	3.03 ± 0.29	3.47 ± 0.39	3.02 ± 0.25	3.46 ± 0.41	0.98	0.20	0.99
Visceral fat	36.15 ± 2.83	28.90 ± 4.26	46.23 ± 3.65	41.93 ± 5.45	0.008	0.17	0.73
Perirenal fat	17.78 ± 1.81	15.44 ± 2.63	20.88 ± 2.04	20.55 ± 2.73	0.086	0.57	0.67

Data are means ± SEM. *P* values for main effect (HFS and WG) or interaction (HFS × WG) were presented in bold numbers when there was a statistically significant (*P* < 0.05) difference. When the HFS × WG interaction was significant (*P* < 0.05) by 2-factor ANOVA, differences between groups are indicated with letters. Means without a common superscript are significantly different (*P* < 0.05) from each other. *n* = 10–12 mice per group.

### WG supplementation improved glucose homeostasis and reduced serum TGs in HFS-fed mice

**FIGURE 1:**
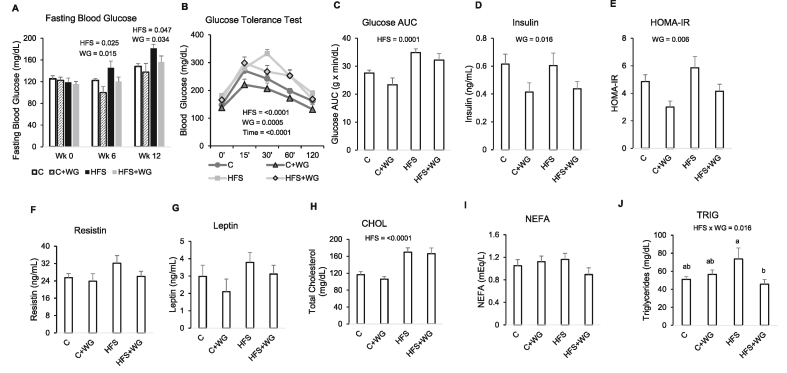
Effects of wheat germ (WG) on metabolic parameters in C57BL/6J mice fed a control (C) or high-fat and sucrose (HFS) diet supplemented with 10% WG for 12 wk. (A) Fasting blood glucose on wk 0, 6, and 12, (B) Glucose tolerance test, (C) Glucose AUC, (D) Fasting serum insulin, (E) HOMA-IR, (F) Serum resistin, (G) Serum leptin, (H) Serum total cholesterol, (I) Serum NEFA, and (J) Serum TGs. Data are means ± SEM. *P* values for main effect (HFS, WG, or Time) were displayed on the figures only if there was a statistically significant (*P* < 0.05) difference. When the HFS × WG interaction was significant (*P* < 0.05) by 2-factor ANOVA, differences between the groups are indicated with letters. Means without a common letter are significantly (*P* < 0.05) different from each other. A-C, H-J (*n* = 10–12 per group). D–G (*n* = 8 per group). AUC, area under the curve; NEFA, nonesterified fatty acids

At wk 6 and wk 12, fasting blood glucose was elevated in the HFS group compared with C by 18% and 23%, respectively (Figure 1A, *P*_*HFS*_ = 0.025 and 0.047 for wks 6 and 12, respectively). On the other hand, WG reduced fasting blood glucose at both time points (*P*_*WG*_ = 0.015 and 0.034 for wks 6 and 12, respectively), as evident in a 17% and 19% decrease in the HFS + WG group compared with HFS at wks 6 and 12, respectively (Figure 1A). The glucose AUC calculated after conducting a GTT showed a modest decrease (≤ −8%, *P*_*WG*_ = 0.079) in glucose AUC due to WG supplementation (Figure 1B–C). Supplemental WG reduced (*P*_*WG*_ *=* 0.016) serum fasting insulin by at least 27% (Figure 1D) and significantly (*P*_*WG*_ = 0.006) improved the insulin resistance marker, HOMA-IR by at least −29% (Figure 1E).

HFS feeding tended to upregulate the VAT leptin (*P*_*HFS*_ = 0.073) and adipose TG lipase (*Pnpla2*, *P*_*HFS*_ = 0.10) genes (Supplemental Figure 1A–B). as well as serum resistin (*P*_*HFS*_ = 0.071, Figure 1F), whereas WG had no significant effect on these parameters. In addition, WG supplementation also had no effect on serum leptin, cholesterol, and nonesterified fatty acids (Figure 1G–I). We observed a significant interaction effect with serum TGs (*P*_HFSxWG_ = 0.016); with HFS + WG reducing serum TGs by 38% compared with HFS (*P* = 0.014) but no effect when added to the control diet (Figure 1J).

### Supplemental WG modulates the jejunum by activating STAT3 and upregulating antimicrobial peptide and anti-inflammatory genes in mice fed an HFS diet

**FIGURE 2:**
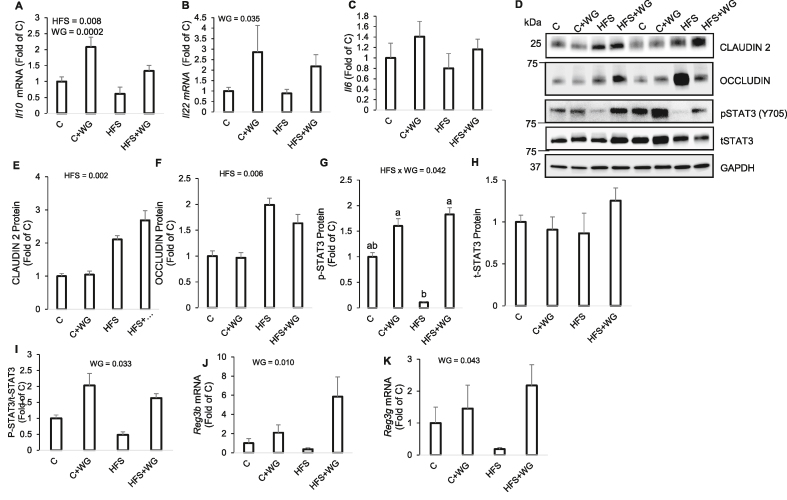
Effects of wheat germ (WG) on jejunal protein and gene expression in C57BL/6J mice fed a control (C) or high-fat and sucrose (HFS) diet supplemented with 10% WG for 12 wk. (A) *Il10* mRNA, (B) *Il22* mRNA, (C) *Il6* mRNA, (D) Representative immunoblots, (E) CLAUDIN 2 protein, (F) OCCLUDIN protein, (G) phosphorylated STAT3, (H) Total STAT3 protein, (I) p-STAT3 / t-STAT3, (J) *Reg3β* mRNA, and (K) *Reg3γ* mRNA expression. Data are means ± SEM. *P* values for main effect (HFS and WG) were displayed on the figures only if there was a statistically significant (*P* < 0.05) difference. When the HFS × WG interaction was significant (*P* < 0.05) by 2-factor ANOVA), differences between the groups are indicated with letters. Means without a common letter are significantly (*P* < 0.05) different from each other. *n* =5–6 per group. Reg3β, regenerating islet-derived protein 3-beta; Reg3γ, regenerating islet-derived protein 3-gamma; STAT3, signal transducer and activator of transcription 3

WG supplementation upregulated (*P*_*WG*_ = 0.0002) the jejunal *Il10* gene by at least 116% (Figure 2A). Similarly, WG showed a main effect of upregulating (*P*_*WG*_ = 0.035) the *Il22* gene by at least 147 % (Figure 2B). Neither WG nor HFS had any effect on *Il6* gene expression (Figure 2C). Feeding an HFS diet resulted in elevated levels of CLAUDIN 2 (≥101%, *P*_*HFS*_ *=*0.002) and OCCLUDIN (≥63%, *P*_*HFS*_ = 0.006) proteins in the jejunum compared with the C diets and WG had no effect on both these proteins (Figure 2D–F). We observed a significant interaction (*P*_HFSxWG_ = 0.042) effect with jejunal STAT3 phosphorylation; with HFS + WG being significantly higher compared with the HFS group but no difference between the C and C + WG groups (Figure 2D, 2G). The dietary treatments did not impact total STAT3 (Figure 2H) but the pSTAT3/t-STAT3 ratio was high (*P*_*WG*_ = 0.033) because of WG supplementation (Figure 2I). Consequently, the jejunal mRNA expression of *Reg3β* and *Reg3γ* was significantly upregulated (≥42%; *P*_*WG*_ ≤ 0.043) by WG supplementation (Figure 2J–K). Similarly, WG tended (*P*_*WG*_ ≤ 0.078) to upregulate *Reg3β* and *Reg3γ* in the ileum (Supplemental Figure 2A, B).

### WG supplementation prevented Nf-κbp65 phosphorylation and downregulated *Il6* and *Lbp* genes in the visceral adipose tissue of HFS-fed mice

**FIGURE 3:**
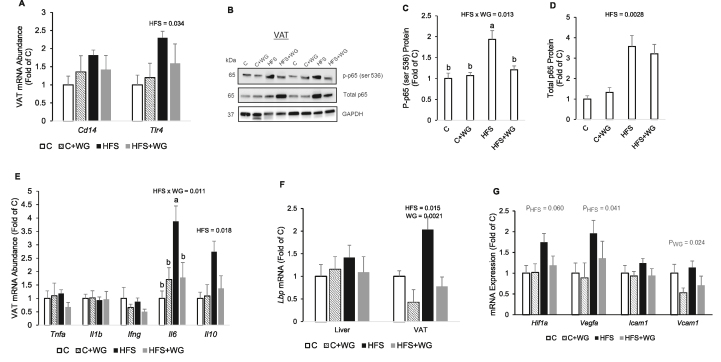
Effects of wheat germ (WG) on inflammatory markers in the visceral adipose tissue (VAT) of C57BL/6J mice fed a control (C) or high-fat and sucrose (HFS) diet supplemented with 10% WG for 12 wk. (A) *Cd14* and *Tlr4* mRNA, (B) Representative immunoblots, (C) phosphorylated NF-κBp65 protein, (D) total NF-κBp65 protein, (E) *Tnfa, Il1b, Ifng, Il6, Il10* mRNA, (F) *Lbp* mRNA in the liver and VAT, and (G) *Hif1a*, *Vegfa*, *Icam1*, and *Vcam1* mRNA. Data are means ± SEM. *P* values for main effect (HFS and WG) were displayed on the figures only if there was a statistically significant (*P* < 0.05) difference., When the HFS × WG interaction was statistically significant (*P* < 0.05) by 2-factor ANOVA, differences between the groups are indicated with letters. Means without a common letter are significantly (*P* < 0.05) different from each other. *n* = 6 per group. Cd14, cluster of differentiation 14; Ifnγ, interferon gamma; LBP, lipopolysaccharide binding protein; Tlr4, toll-like receptor 4; Tnfα, tumor necrosis factor-alpha

At the end of the dietary treatment, neither HFS nor WG had any effect on *Cd14* mRNA (Figure 3A). HFS feeding upregulated (*P*_*HFS*_ *=* 0.034) the *Tlr4* gene in the VAT by ≥60% whereas WG had no effect (Figure 3A). We observed a significant interaction (*P*_HFSxWG_ = 0.013) on the phosphorylated NF-κBp65 protein (Figure 3C). The activation of NF-κBp65 protein was elevated (*P =* 0.0019) by 94% in the HFS group compared with the control (Figure 3B, C). Interestingly, WG supplementation in the HFS diet significantly suppressed (*P =* 0.014) the HFS-induced activation of NF-κBp65 by 38%, thus, bringing it to the level of control (Figure 3B, C). There was a main effect of HFS (*P*_*HFS*_ = 0.0028) in elevating total NF-κBp65 protein in the VAT; (Figure 3B, D).

We also observed significant interaction (*P*_HFSxWG_ = 0.011) effects on the *Il6* gene which was reflected in a 3-fold increase of the *Il6* mRNA in the HFS group compared with the C group (*P =* 0.0032), whereas the HF S+ WG group downregulated *Il6* gene by 54% compared with the HFS group (*P =* 0.034; Figure 3E). Moreover, HFS feeding significantly upregulated (*P*_*HFS*_ *=* 0.018) the *Il10 gene*. Interestingly, the expression of the *Lbp* gene was signifcantly upregulated by HFS (*P*_*HFS*_ = 0.015), whereas WG supplementation showed a main effect (*P*_*WG*_ *=* 0.0021) to attenuate *Lbp* expression in VAT (Figure 3F). Dietary treatments had no significant effects on *Lbp* gene (Figure 3F) and other inflammatory gene markers in the liver (Supplemental Figure 3A–E). Finally, WG significantly suppressed (*P* = 0.024) *Vcam1* gene in the VAT but had no effect on the HFS-induced upregulation of *Hif1a* and *Vegfa* (Figure 3G).

### Supplemental WG suppressed innate and adaptive immune cells markers in the visceral adipose tissue of HFS-fed mice

**FIGURE 4:**
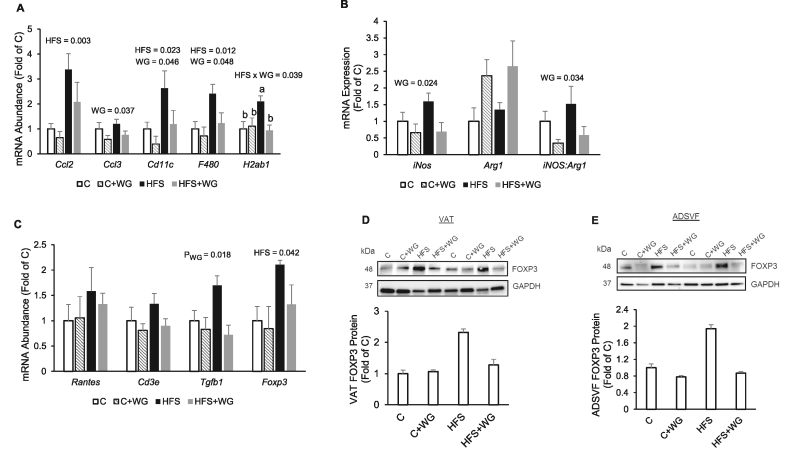
Effects of wheat germ (WG) on macrophage and T-cell related markers in the visceral adipose tissue (VAT) and adipose-derived stromal vascular fraction **(**ADSVF) of C57BL/6J mice fed a control (C) or high-fat and sucrose (HFS) diet supplemented with 10% WG for 12 wk. (A) VAT *Ccl2, Ccl3, Cd11c, F4/80, H2ab1* mRNA, (B) VAT mRNA expression of *iNos, Arg1*, and *iNOS-Arg1* mRNA expression ratio, (C) VAT mRNA expression of *Rantes, Cd3e, Tgfb1, Foxp3*, (D) VAT FOXP3 protein expression, and (E) FOXP3 protein expression in the ADSVF. Data are means ± SEM. *P* values for main effect (HFS and WG) were displayed on the figures only if there was a statistically significant (*P* < 0.05) difference. When the HFS × WG interaction was significant (*P* < 0.05) by 2-factor ANOVA, differences between groups are indicated with letters). Means without a common letter are significantly (*P* < 0.05) different from each other. A-D (*n* = 6 per group), E (*n* = 5 per group). Arg1, arginase 1; Cd3e, cluster of differentiation 3e; Ccl2, C-C motif chemokine ligand 2; Foxp3, forkhead box protein 3, iNos, inducible nitric oxide synthase; Tgfβ1, transforming growth factor beta

As presented in Figure 4A, HFS feeding elevated (*P*_*HFS*_ *=* 0.0026)*,* VAT mRNA expression of the chemokine *Ccl2* (≥1.5-fold) but not *Ccl3.* On the other hand, WG supplementation showed a trend (*P*_*WG*_ *=* 0.10) to decrease *Ccl2* gene and significantly reduced (*P*_*WG*_ *=* 0.037) *Ccl3* gene expression (Figure 4A). The gene expression of the macrophage and dendritic cell marker, *Cd11c*, and the macrophage marker *F4/80* in VAT were significantly (*P*_*HFS*_ *≤* 0.023) elevated by at least 141% by HFS feeding, whereas WG supplementation downregulated (*P*_*WG*_ *≤* 0.048) the expression of these genes by at least 28%; (Figure 4A). We observed a significant interaction effect (*P*_HFSxWG_ = 0.039) on the antigen presentation gene marker, *H2ab1*, which encodes the major histocompatibility complex 2 (MHC II;Figure 4A). The *H2ab1* gene was significantly upregulated (1-fold, *P =* 0.012) in the HFS group compared with the C, whereas HFS + WG significantly repressed (−55%; *P =* 0.013) the expression of this gene compared with the HFS group (Figure 4A).

Furthermore, WG supplementation significantly downregulated (*P*_*WG*_ *=* 0.024) the mRNA expression of the MI macrophage marker, *iNos*, by at least 34% while it also showed a tendency (*P*_*WG*_ *=* 0.086) to increase the gene expression of *Arg1*, an M2 macrophage marker (Figure 4B). The ratio of *iNos* to *Arg1* gene expression was significantly decreased (≥ −65%; *P*_*WG*_ *=* 0.034) by supplemental WG; (Figure 4B). Moreover, data in Figure 4C showed that the dietary treatments had no significant impact on the VAT gene expression of the T-cell marker, *Cd3e,* and the T-cell attractant, *Rantes* (Figure 4C). However, we observed a significant (*P*_*WG*_ *=* 0.018) WG effect on the mRNA abundance of *Tgfb1* (Figure 4C). HFS feeding significantly upregulated (*P*_*HFS*_ *=* 0.042) *Foxp3* mRNA (Figure 4C) and a tendency (*P*_*WG*_ *=* 0.058) to upregulate the protein expression of FOXP3 in the VAT (Figure 4D). WG showed a tendency (*P*_*WG*_ *=* 0.058) to reduce FOXP3 protein in the ADSVF (Figure 4E).

## Discussion

Diet-induced obesity (DIO) is associated with a leaky gut in both animals and humans, which increases chronic bacteremia and endotoxemia capable of initiating systemic inflammation and insulin resistance via the activation of the TLR4-NF-κB pathway [5, 37]. Using a Western diet-induced (i.e., HFS) model of obesity, this study investigated the gut-protective mechanism of WG supplementation and the implication on inflammation and immune cell infiltration in the VAT of C57BL/6J mice fed a control or a Western diet for 12 wk. Although WG had no effect on tight junction proteins in the jejunum, our study demonstrated that WG activates the STAT3 pathway and upregulates AMP genes (Reg3β and Reg3γ) in the jejunum. Consequently, WG attenuated NF-κB phosphorylation in the VAT, together with the down-regulation of *Il6* and *Lbp* genes. In addition, WG supplementation resulted in fewer chemoattractant and macrophage gene markers in the VAT at the end of dietary treatment.

Obesity-induced metabolic syndrome is characterized in part, by insulin resistance, increased abdominal adiposity, and dyslipidemia [38]. In mice, various HF diet compositions have been used to model obesity-induced metabolic syndrome. The degree of response of these animals to HF diets depends on several factors, including duration and genetic background [39]. In this study, we only observed significant HFS effects on few markers of obesity-induced metabolic syndrome, including increased visceral fat, elevated fasting blood glucose (∼181 mg/dL), and glucose intolerance compared with the C diet. In addition, HFS showed modest effects on elevating total body fat, HOMA-IR, fasting TGs, and the adipokines, leptin, and resistin. Supplemental WG had the opposite effect in most cases because it significantly reduced fasting glycemia, fasting insulin, HOMA-IR, and serum TGs, especially in the HFS-fed mice. Considering that the fasting blood glucose of insulin resistant C57BL/6J mice may be >240 mg/dL [40], the overall implication of the metabolic outcomes of the HFS-fed C57BL/6J mice in our study, especially as it relates to glucose metabolism, suggest that these animals are likely in the early stages of developing obesity-induced insulin resistance. Nevertheless, WG supplementation showed a strong potential to improve glucose metabolic parameters.

DIO impairs the expression of TJ proteins and the normal architecture of the gut epithelial TJ in both animals and humans [41]. As a result, bacteremia and endotoxemia ensue that is associated with the initiation of inflammation in peripheral tissues and insulin resistance [5, 42]. Although the transcellular pathway allows microbial components through the epithelium by endocytosis [43, 44], elevated expression of the pore-forming CLAUDIN 2 is also associated with the leaky gut phenomenon in various diseases [15], including in DIO studies [16, 17]. Accordingly, we observed an increase in CLAUDIN 2 expression in the jejunum of HFS mice while WG had no effect. This suggests an increased potential of bacteremia and endotoxemia in the HFS group that could be vital for the initiation of inflammation in peripheral tissues, such as the adipose tissue. This notion is in agreement with the finding that HF feeding induces consistent paracellular permeability over time in the rat jejunum [18]. Suprisingly, HFS feeding also increased the protein level of OCCLUDIN in the jejunum which may be a partial compensatory action to restore the jejunal TJs as observed in another rat study [45]. However, this compensatory mechanism may not necessarily be beneficial or efficient in preventing a leaky gut, as elevated OCCLUDIN in obese animals was observed to be localized in the cytoplasm rather than the apical cellular border [45, 46].

To reduce bacterial-epithelial interaction and mitigate gut translocation of microbes, AMPs play a main role by reducing bacterial density within the mucosal layer [23, 47]. Among the AMPs, Reg3β and Reg3γ have been reported for their potent bactericidal action against gram-positive and gram-negative bacteria [47, 48]. In the gut, butyrate produced from bacterial fermentation of dietary fiber activates STAT3 in the epithelium resulting in the generation of the Reg3 lectins [22]. Furthermore, indole metabolites, derived from tryptophan metabolism by *Lactobacillus*, stimulate the production of IL-22 from lamina propria lymphocytes capable of activating the STAT3-Reg3 pathway [21, 49, 50]. We have previously reported that WG supplementation in HFS-fed mice selectively increased *Lactobacillus* but did not increase fecal SCFAs [26]. In the present study, WG upregulated IL-22 mRNA expression and increased jejunal STAT3 phosphorylation. Consequently, we observed a WG-induced increase in the abundance of Reg3 genes (Reg3β and Reg3γ) in the jejunum. Considering that WG has a modest fiber content (3%–4%) but is a rich source of tryptophan (up to 300 mg per 100 g WG) [[28], [29], [30]], it is plausible that the potential of WG to increase *Lactobacillus* in HF-fed mice resulted in indole metabolites that activated the IL-22–STAT3 pathway, resulting in elevated Reg3 AMPs. The AMPs may then be vital to reducing the burden of antigen translocation that could initiate inflammation and insulin resistance in peripheral tissues. Future studies should measure these indole metabolites to ascertain which is responsible for activating the IL-22-STAT3 pathway and elevating Reg3 AMPs.

Accordingly, our study revealed the ability of WG to modulate adipose tissue inflammation, a classic response in DIO. First, we observed an HFS diet-induced increase in total NF-κBp65 in the VAT similar to other rodent studies [[51], [52], [53]]. Although WG had no effect on total NF-κBp65, supplemental WG attenuated HFS-induced phosphorylation of NF-κBp65, a key component of the NF-κB complex that drives macrophage recruitment and the transcription of several proinflammatory genes [54]. Consequently, WG suppressed the HFS-induced upregulation in *Il6, Lbp*, and macrophage-related genes such as *Cd11c* and *F4/80* in the adipose tissue. By recognizing the lipid A moiety of LPS, a cell wall component of gram-negative bacteria, an LBP-LPS complex is formed that increases the sensitivity of Cd14/TLR4-NF-κB pathway to LPS by several folds [9, 10, 12]. Furthermore, adipose-derived LBP directs local inflammatory and metabolic responses and may be an early biomarker for adipose tissue dysfunction in obesity [11]. Put together, the ability of WG to upregulate AMPs in the jejunum in this study may be vital for the prevention of NF-κBp65 activation and attenuation of *Il6* and *Lbp* genes in VAT of mice fed an HFS diet.

In addition, it was intriguing to observe an HFS-induced increase in the anti-inflammatory gene, *Il10*, in the VAT. The cell population that could contribute to the IL-10 pool in the adipose tissue include macrophages (M2) and T-regulatory (FoxP3+) cells [3]. However, WG but not HFS tended to increase the M2 macrophage marker gene - *Arg1* in the VAT. On the other hand, the *FoxP3* gene and protein in the adipose tissue and the stromal vascular fraction showed a strong trend to be higher in the HFS group than in the others. Overall, this is in agreement with studies that show elevated adipose IL-10 or FoxP3 in obese animals and humans, suggesting a compensatory effect on the proinflammatory environment [[55], [56], [57], [58]]. In addition, the initial increase in VAT FOXP3-expressing cells in obese animals progressively reduced over time, which worsened inflammatory and metabolic outcomes [57]. It should be noted that the metabolic parameters from our HFS-fed mice suggest that these animals are at the early stages of developing obesity-induced insulin resistance. Thus, our finding of elevated VAT *Il10* and *FOXP3* genes in the HFS-fed group may be an initial compensatory action that aimed to ameliorate the consequence of the leaky gut-associated production of proinflammatory factors observed also in the HFS group. Importantly, supplemental WG showed no effect to elevate these anti-inflammatory factors unlike HFS, due to the less need to combat HFS-induced inflammation.

Although the findings of this study are exciting, it is not without limitations. Some of the limitations include the use of a powdered diet, which makes it hard to accurately monitor food intake that can affect metabolic outcomes, including body weight and body composition. We also did not measure the amount of tryptophan in our diets, and we cannot assess the contribution of the gut bacterial-derived indole metabolites from tryptophan metabolism. Additionally, it is still unclear whether the effect of WG on STAT3 reported in this study is solely via IL-22. Butyrate was not elevated in HFS-fed mice supplemented with WG [26]; hence, the direct effect of WG-induced butyrate on the STAT3 pathway is unlikely. However, IL-10 is also capable of activating STAT3 in certain models of disease [59, 60]. Because supplemental WG also increased *Il10* gene in the jejunum in this study, it is probable that the IL-10 pathway contributes to STAT3 activation in the jejunum. Nevertheless, our study suggests that whole foods, such as WG may be gut-protective in HF feeding by various synergistic mechanisms. Therefore, future studies may find it important to clarify these individual mechanisms.

In conclusion, this study showed that WG, activates the STAT3 pathway and upregulates the *Il22* and AMP genes (*Reg3b* and *Reg3g*) in the jejunum in C57BL/6J mice that fed an HFS diet for 12 wk. Accordingly, WG attenuated HFS-induced upregulation of *Il6* and *Lbp* genes, and lessened NF-κBp65 phosphorylation in the visceral adipose tissue to the level of C. In addition, supplemental WG improved metabolic parameters in HFS-fed animals, including fasting blood glucose and insulin resistance marker (HOMA-IR). Put together, the gut modulatory effects of WG on STAT3 and AMPs may be vital to reduce the burden of obesity-induced adipose tissue inflammation and diet-induced insulin resistance. However, the effects of WG in increasing lean mass may also contribute to the favorable changes in insulin resistance markers independent of its effects on VAT inflammation.

## Author disclosures

BAO, SEA, AK, SYW, BK, JR, WC, DL, SC, BJS, and EAL, have conflicts of interest.

## Data Availability

The data for this article are available in the article and in its online supplementary material. Any additional data will be shared on reasonable request to the corresponding author

## Appendix A Supplementary data

Multimedia components 1 Multimedia components 1
