# Supplementary material for: Supplemental wheat germ modulates phosphorylation of STAT3 in the gut and NF-κBp65 in the adipose tissue of mice fed a Western diet
Source: Curr Dev Nutr. 2022 Dec 23;7(1):100023. doi: 10.1016/j.cdnut.2022.100023 (PMC10100941; doi:10.1016/j.cdnut.2022.100023)

Online Supporting Material

Supplemental Table 1: Primer Sequence for Gene Expression Analyses

Gene	Forward	Reverse
mArg1	5'-cagtctggcagttggaagca-3`	5'-gcatccacccaaatgacaca-3`
mCcl2	5'-cttcctccaccaccatgca-3`	5'-ccagccggcaactgtga-3`
mCcl3	5`-ttcatcgttgactattttgaaacca-3`	5`-gccggtttctcttagtcaggaa-3`
mCd3e	5`-tagccagtgctgggacattg-3`	5`-caatctcagcagcctgattcttt-3`
mCd11c	5'-cttcattctgaagggcaacct-3`	5`-cactcaggagcaacaccttttt-3`
mCd14	5'-gccgccaccgcttct-3`	5'-acacgttgcggaggttca-3`
mCyclo	5`-tgg agagca cca aga cag aca-3`	5`-tgc cgg agt cga caa tga t-3`
mF4/80	5`-tggccaagattctcttcctcac-3`	5`-gcctccactagcatccagaaga-3`
mFoxp3	5`-ggcccttctccaggacaga-3`	5`-ggcatgggcatccacagt-3`
mH2ab1	5`-gcctgaagagccccatcac-3`	5`-tgccgctcaacattttgct-3`
mHif1a	5'-caacgtggaaggtgcttca-3`	5'-tgaggttggttactgttggtatca-3`
mIcam1	5'-ggaggtggcgggaaagtt-3`	5'-tccagccgaggaccatacag-3`
mIfng	5`-atgaacgctacacactgcatc-3`	5`-ccatccttttgccagttcctc-3`
mIl1b	5'-caaccaacaagtgatattctccatg-3'	5'-gatccacactctccagctgca-3`
mIl10	5`-ggt tgc caa gcc tta tcg ga-3`	5`-acc tgc tcc act gcc ttg ct-3`
mIl6	5`-gag gat acc act ccc aac aga cc-3`	5`-aag tgc atc atc gtt gtt cat aca-3`
mIl22	5`-atgagtttttcccttatggggac-3`	5`-gctggaagttggacacctcaa-3`
miNos	5'-caggaggagagagatccgattta-3`	5'-gcattagcatggaagcaaaga-3`
mLbp	5`-gtcctgggaatctgtccttg-3`	5`-ccggtaaccttgctgttgtt-3`
mLep	5`-ctccatctggccttctc-3`	5`-catccaggctctctggcttct-3`
mPnpla2	5`-aaggacctgatgaccaccct-3`	5`-ccaacaagcggatggtgaag-3`
mRantes	5'-ggagtatttctacaccagcagcaa-3`	5'-ggcggttccttcgagtga-3`
mReg3β	5`-tgg gaa tgg agt aac aat g-3`	5`-ggc aac ttc acc tca cat-3`
mReg3γ	5`-cca tct tca cgt agc agc-3`	5`-caa gat gtc ctg agg gc-3`
mTgfb1	5`-ctcccgtggcttctagtgc-3`	5`-gccttagtttggacaggatctg-3`
mTlr4	5'-actgttcttctcctgcctgaca-3`	5'-tgatccatgcattggtaggtaata-3`
mTnfa	5`-ctgaggtcaatctgcccaagtac-3`	5`-cttcacagagcaatgactccaaag-3`
mVcam1	5`-tgaacccaaacagaggcagagt-3`	5`-ggtatcccatcacttgagcagg-3`

mVegfa	5`-cgctggtagacgtccatga-3`	5`-cacgacagaaggagagcagaa-3`

4

<u>Supplemental Figure 1</u>

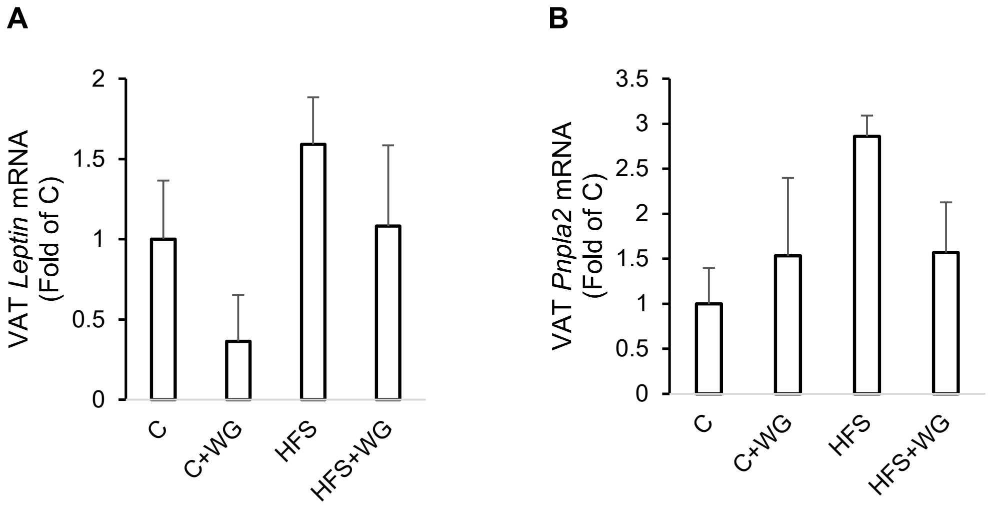

Supplemental Figure 2

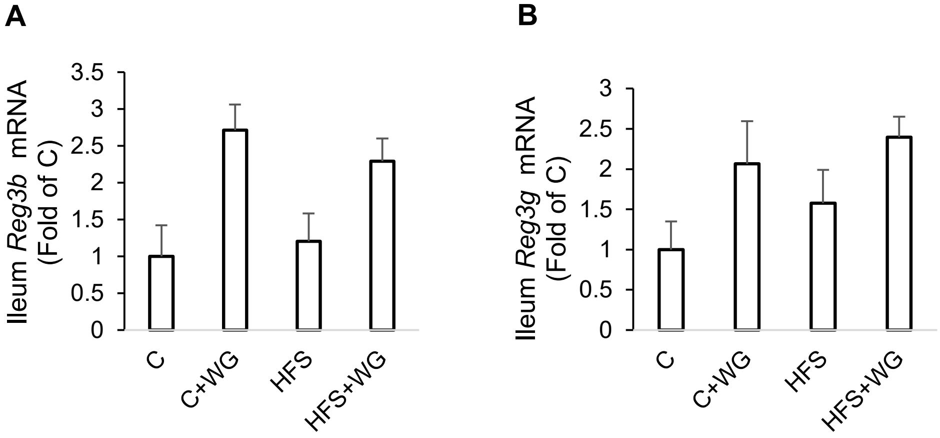

Supplemental Figure 3: Liver

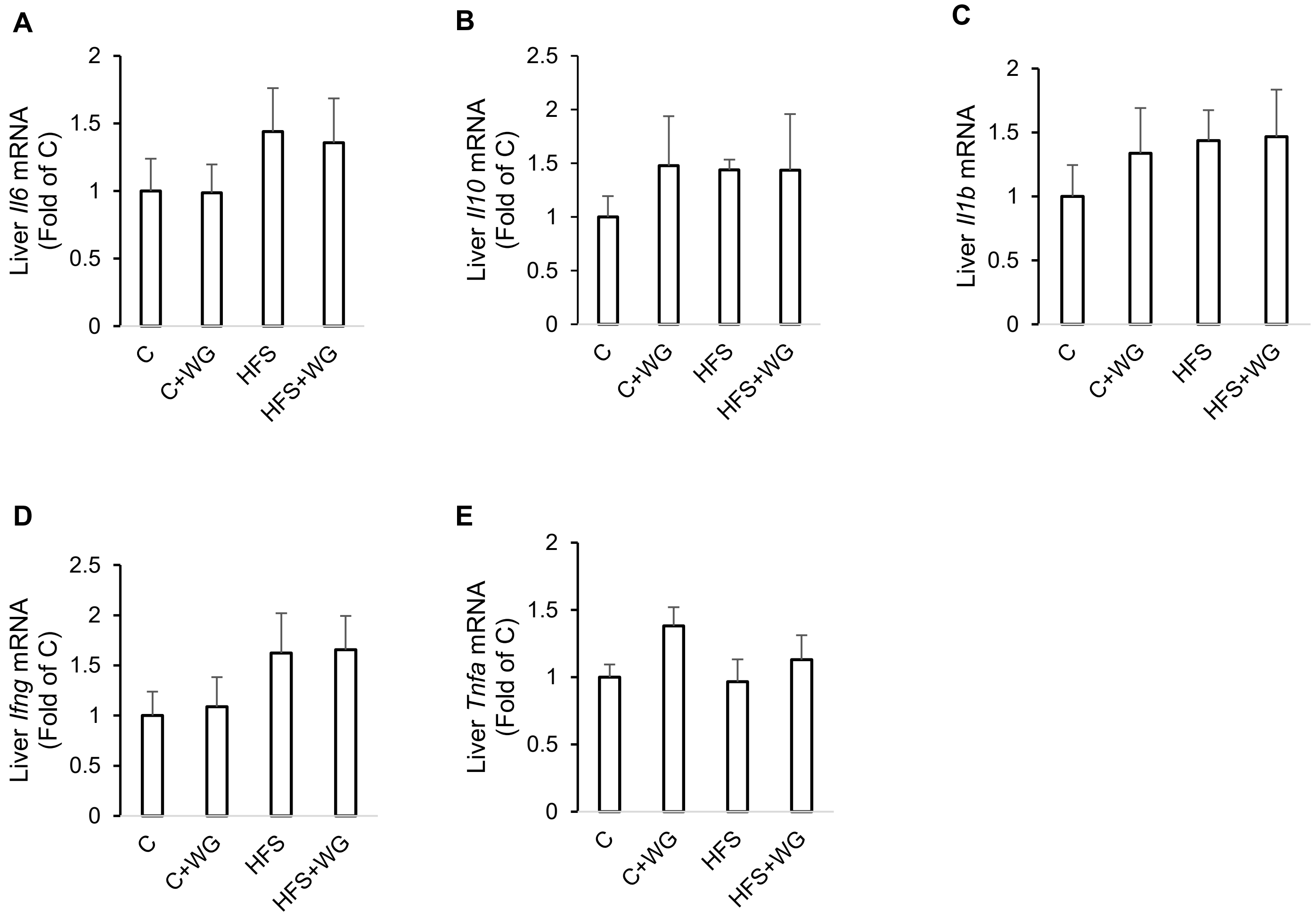

Supplemental Figure 4: VAT

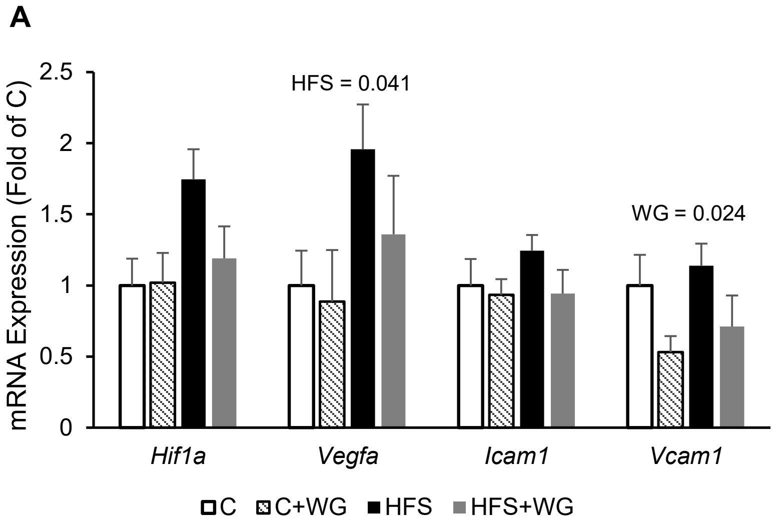

Supplement: Multimedia components 1 [file mmc1.pdf]
